# Effects of a traditional herbal medicine on peripheral blood flow in women experiencing peripheral coldness: a randomized controlled trial

**DOI:** 10.1186/s12906-015-0617-4

**Published:** 2015-04-02

**Authors:** Shinji Nishida, Eri Eguchi, Tetsuya Ohira, Akihiko Kitamura, Yukiko Hakariya Kato, Keisuke Hagihara, Hiroyasu Iso

**Affiliations:** Department of Psychosomatic Medicine, Japanese Red Cross Wakayama Medical Center, Wakayama, Japan; Department of Kampo Medicine, Osaka University Graduate School of Medicine, Suita, Japan; Department of Public Health, Okayama University Graduate School of Medicine, Dentistry, and Pharmaceutical Sciences, Okayama, Japan; Department of Epidemiology, Fukushima Medical University, Fukushima, Japan; Public Health, Department of Social Medicine, Osaka University Graduate School of Medicine, Suita, Japan; Osaka Center for Cancer and Cardiovascular Diseases Prevention, Osaka, Japan; Takarazuka City Health Promotion Center, Takarazuka, Japan

## Abstract

**Background:**

In Japan, a traditional herbal medicine, *Tokishigyakukagoshuyushokyoto* (TJ-38), is often used for the treatment of peripheral coldness, which is a common complaint among Japanese women. However, the effects of this herbal medicine have yet to be examined in a randomized controlled trial. In the current study, the effect of TJ-38 on the peripheral blood flow in women experiencing peripheral coldness was investigated using a parallel-group randomized controlled trial.

**Methods:**

Fifty-eight women aged 23 to 79 years with peripheral coldness were randomly divided into the intervention or control group. They were examined using cold bathing tests, physical examinations, and questionnaires in January 2010 for the baseline and in March 2010 for the follow-up, and January 2011 and March 2011, respectively.

**Results:**

At the baseline, there were no differences in clinical characteristics between the two groups. In the intervention group, peripheral coldness improved after the intervention term; however, it persisted in the control group. Mean values of percentage recovery of the peripheral blood flow after cold bathing tests were 17.2% and −28.2% for the intervention and control groups, respectively (p = 0.007), and the proportions for percentage recovery of >50% were 32% and 0%, respectively (p = 0.0007). Mean values of percent recovery of skin temperature did not differ between the two groups.

**Conclusions:**

The present clinical trial supports that a traditional herbal medicine relieves peripheral coldness in women probably through the improvement of peripheral blood flow.

## Background

Peripheral coldness is a common symptom in Japanese women, often complicating other symptoms such as headaches, depressive moods, and fatigue [1-3]. Whilst it is not a fatal disease, it can lead to a decreased quality of life [4]. In previous studies [2,5], peripheral coldness was observed in 39% of female university officials and 52% of women who visited a gynecological clinic for regular checkups, some of whom had gynecological or menopausal complaints. The etiology of peripheral coldness is not well established, but it could be associated with decreased peripheral blood flow. Women with peripheral coldness in both the upper and lower extremities had about 50% lower blood flow (as measured by a laser Doppler meter) in the upper extremities and about 60% lower blood flow in the lower extremities as compared to those without peripheral coldness [6]. Another study of 41 women with and without peripheral coldness showed lower skin temperatures of the palms and feet, as well as a larger temperature range in women with peripheral coldness, as compared to those without peripheral coldness [7]. Furthermore, women with peripheral coldness tend to have a lower body mass index and less body fat than those without it [8]. In Japan, more than 20% of women aged 20-29 years are underweight (BMI 18.5 kg/m^2^) [9], and that percentage was much higher than that in most developed countries, highlighting the importance of effective treatment for peripheral coldness.

In Japan, peripheral coldness is commonly treated by vitamin E and lifestyle modification. However, the effectiveness of these treatments has been questioned [10-12]. The administration of traditional herbal medicine is one of the alternative approaches in the treatment of peripheral coldness, and has been reported to reduce symptoms. *Tokishigyakukagoshuyushokyoto* (TJ-38) is a traditional herbal medicine originally from Chinese medicine and now used widely in Japan. TJ-38 is one of commonly used traditional herbal medicine in Japan because it contains vasodilators and anticoagulant ingredients such as evodiame, adenosine, ligustilide and butylidenephthalide [13,14]. Previous case-analysis studies reported that TJ-38 improved patient’s symptoms associated with peripheral coldness. A study with 181 patients with peripheral coldness showed that the administration of TJ-38 improved 74% of participants’ peripheral coldness [15]. Also, another case-report study showed that patient’s peripheral coldness decreased 4 days after the start of TJ-38 administration [16]. However, no randomized controlled clinical trial has been conducted to prove the effectiveness of TJ-38 in adult women. Furthermore, the mechanism of reducing peripheral coldness is unknown. Therefore, the purpose of this study was to investigate, through a parallel group designed randomized controlled trial, the effect of a traditional herbal medicine on peripheral blood flow, skin temperature and subjective symptoms in women with peripheral coldness.

## Methods

### Ethics statement

Written informed consent was obtained from all participants. The study had been approved by the Ethics Committee of the Osaka University Graduate School of Medicine on December 17th, 2009 (No. 09152). The authors confirm that all ongoing and related trials for this drug/intervention are registered.

### Inclusion and exclusion criteria

We included women aged 20 to 80 years who had experienced peripheral coldness for at least three years. The exclusion criteria were pregnancy or any peripheral circulatory disorders such as advanced diabetes mellitus, Buerger’s disease, hypothyroidism, hyperthyroidism, and connective tissue disorders.

### Participants

Participants of this study were recruited via websites, bulletin board postings, flyers, and newspaper advertisements from October 22 to December 10 in 2009, and November 5 to December 20 in 2010. Women interested in the study attended explanatory seminars at Osaka University Hospital and Osaka Medical Center for Health Science and Promotion (currently Osaka Center for Cancer and Cardiovascular Disease Prevention). In total, 75 women contacted the study office, and 58 women aged 23 to 79 years met the inclusion criteria and agreed to participate in the study with written informed consent.

### Trial design

The design of the trial in this study was a parallel-group randomized controlled trial. Participants were randomly divided into intervention and control groups, and stratified by age (<50 and ≥50). The allocation of the intervention and control groups was performed using random numbers. Concealed random numbers ranging from 0 to 1 on an Excel file were used to determine the groups, with a cutoff value of 0.5. The study office administrative staff dealt with the enrolment of the participants and automatically assigned them to two groups based on the Excel file. The allocation ratio for intervention and control groups was 1:1 each year. In the trial, the researchers who assessed outcomes did not know the allocated group of each subject, but the subjects knew which group they were allocated to. After randomization, 28 subjects were included in the intervention group and 30 in the control group. The sample size was determined by sample size calculation. It was assumed that approximately 30% of the women would have an improved percentage recovery of blood flow after the intervention, while approximately 0% (1% for calculation purposes) of the women would have improved in the control group. The result of the power calculation was 87.1% with 28 subjects for each group.

The baseline and the eight-week follow-up examinations were held at the Osaka Medical Center for Health Science and Promotion on January 5 to 13 and March 1 to 10 in 2010, respectively, and on January 5 to 11 and March 1 to 7 in 2011, respectively. In February 2010 and 2011, a physician monitored the participants’ health status, daily symptoms of peripheral coldness, and gave lifestyle guidance on the management of peripheral coldness for both intervention and control groups, and monitored side-effects for intervention group. Participants paid for their own prescribed traditional herbal medicine, and all participants received 10,000 yen after the trial.

### Interventions

Women in the intervention group were administered 7.5 g of a traditional herbal medicine, TSUMURA & CO. *Tokishigyakukagoshuyushokyoto* (TJ-38), per day (2.5 g, three times a day) for eight weeks from January to March, between the baseline and follow-up examinations. The intake status of TJ-38 was recorded by the participants and it was confirmed in the midpoint of intervention by a physician. During the trial, two women in the intervention group complained of a light stomach ache and their dose of TJ-38 was reduced to 2.5 g/day for the rest of the intervention period (two weeks). As the symptoms disappeared after reducing the TJ-38 dose, these two women were included in the analysis. No other adverse effects were observed during the trial. Subjects in the control group were asked to have a usual life, and only received a lifestyle guidance on the management of peripheral coldness.

TJ-38 is a traditional herbal medicine in the form of a granulated extract. A 7.5 g dose of TJ-38 contains 4.0 g of a dried, water extracted mixture of nine crude drugs, with magnesium stearate and lactose hydrate as additives. The proportions of the nine crude drugs are as follows: 5.0 g of jujube, 3.0 g of cinnamon bark, 3.0 g of peony root, 3.0 g of Japanese angelica root, 3.0 g of akebia stem, 2.0 g of glycyrrhiza, 2.0 g of evodia fruit, 2.0 g of asiasarum root, and 1.0 g of ginger. Detailed information regarding to these nine crude drugs are shown in Table 1. The original plants and the quality of these crude drugs are regulated by the Japanese Pharmacopoeia.

**Table 1:** **Information on nine crude drugs composed of Tokishigyakukagoshuyushokyoto (TJ-38)**

**English**	**Latin**	**Japanese**	**Chinese**	**Dosage (g)**	**Place of origin**
Jujube	Zizyphi Fructus	Taisou	da zao	5.0	China
Cinnamon bark	Cinnamomi Cortex	Keihi	gui pi	3.0	China
Peony root	Paeoniae Radix	Shakuyaku	shao yao	3.0	Japan
Japanese Angelica root	Angelicae Radix	Touki	dang gui	3.0	Japan
Akebia stem	Akebiae Caulis	Mokutsu	mu tong	3.0	Japan
Glycyrrhiza	Glycyrrhizae Radix	Kanzou	gan cao	2.0	China
Evodia fruit	Euodiae Fructus	Goshuyu	wu zhu yu	2.0	China
Asiasarum root	Asiasari Radix	Saishin	xi xin	2.0	China
Ginger	Zingiberis Rhizoma	Shoukyou	sheng jiang	1.0	China

All of the nine crude drugs are under quality control based on 16th edition of the Japanese pharmacopoeia.

### Measurements

In the physical examination, we measured the subjects’ height in stockinged feet and weight in light clothing by using an automated measuring instrument. Body mass index was calculated as weight (kg)/height (m)^2^. Systolic and diastolic arterial blood pressures were measured twice by trained nurses using a mercury sphygmomanometer on the right arm of the participants, in a sitting position after five minutes of rest. The average of the two blood pressure measurements was used for analysis.

Blood was drawn at fasting and the serum was separated after centrifugation to measure serum total cholesterol, LDL-cholesterol, HDL-cholesterol, triglycerides, hs C-reactive protein. The whole blood was used to measure red blood cell counts, hemoglobin, and white blood cell counts.

### Peripheral blood flow and skin temperature

The cold bathing test was performed in a room in which the temperature was maintained at 24 ± 1°C. After a 20 minute equilibrium period, participants placed both hands into water at 4°C for 30 seconds. The peripheral blood flow and skin temperature were measured before submergence and then 1 and 10 minutes after the test. Hands were wiped before those measurements. The peripheral blood flow in the middle finger of the right hand was measured using a laser Doppler device (Periflux Systems System 4000, Perimed, USA). The signal processor of this device establishes a linear relationship between the flow meter output signal and the blood flow for all flow rates. The performance of the signal processor was evaluated through an experimental fluid model, which optically resembles the blood flow through the microvasculature [17]. The skin temperature of the right hand was measured by thermography (Infra Eye 2000B, Nihon Koden, Japan).

The percentage recovery of peripheral blood flow or skin temperature was calculated with the following formula:

$$ \begin{array}{l}\mathrm{Percentage}\ \mathrm{recovery}\ \mathrm{of}\ \mathrm{peripheral}\ \mathrm{blood}\ \mathrm{flow}\ \mathrm{or}\ \mathrm{skin}\ \mathrm{temperature}\\ {}=\frac{\mathrm{the}\kern0.5em \mathrm{value}\kern0.5em 10 \min \kern0.5em \mathrm{after}\kern0.5em \mathrm{test}-\mathrm{the}\kern0.5em \mathrm{value}\kern0.5em \mathrm{before}\kern0.5em \mathrm{test}}{\mathrm{the}\kern0.5em \mathrm{value}\kern0.5em \mathrm{before}\kern0.5em \mathrm{test}-\mathrm{the}\kern0.5em \mathrm{value}\kern0.5em 1\kern0.5em \mathrm{after}\kern0.5em \mathrm{test}}\end{array} $$

Women in both the intervention and control groups also filled out a self-administered questionnaire for the baseline and follow-up examinations, which included items on the symptoms of coldness in the fingers, feet, back, and whole body, lifestyle behaviors, and past illness. The question about perceived coldness was: “In which part of the body do you feel chilliness?”, and the selectable answers (may be multiple) were: “fingers”, “feet”, “back”, and/or “whole body”. In the questionnaire after the intervention period, participants were asked an additional question regarding perceived improvement of coldness symptoms: “Did your symptoms of coldness improve?” for which the selectable answers were: “substantially improved”, “somewhat improved”, “unchanged”, or “deteriorated”. For the analysis, we categorized the “substantially improved” and “somewhat improved” as improved, and “not changed” and “deteriorated” as not improved.

Lifestyle behaviors such as smoking, alcohol intake, exercise habits, and perceived mental stress were assessed. As for smoking, current smokers were defined as persons who smoked at least one cigarette per day for the past three weeks. Alcohol intake was measured in “go” (a traditional Japanese unit of volume corresponding to 23 g of ethanol) per week, which was afterwards converted to grams of ethanol per day. One *go* is equivalent to180 ml of sake, one bottle (500 ml) of beer, two single shots (60 ml) of whiskey, or two glasses (240 ml) of wine. Exercise habits were defined as exercising for 15 or more minutes a week for more than the past three months. The question for perceived mental stress was: “Do you experience stress in your job or your daily life?”, for which the answers were “quite a lot”, “a lot”, “somewhat”, and “a little”. We defined “quite a lot” and “a lot” as positive for perceived mental stress. Use of medication for hypertension, menopausal status, and other aspects of health and symptoms during the past month were also inquired by interviewers.

### Statistical analysis

**Figure 1:**
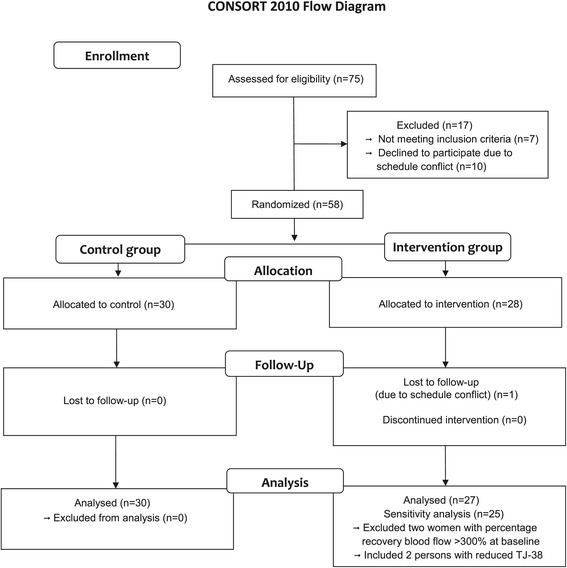
**Flow-chart of participants.** Flow-chart of participant selection in the randomized controlled trial using *Tokishigyakukagoshuyushokyoto* (TJ-38).

One woman in the intervention group could not participate in the follow-up examination because of a schedule conflict (Figure 1). Therefore, the results included 27 interventions and 30 controls for follow-up examination and analysis.

Mean values and proportions of baseline characteristics among the intervention and control groups were calculated, and t-tests were conducted for means, and chi-square tests for proportions of characteristics. The t-tests were also conducted to evaluate the difference in mean values of peripheral blood flow and skin temperature between the intervention and control groups, and chi-square tests to evaluate the difference in the proportion of women whose percentage recovery of blood flow after 10 min was >50% at the baseline and at the eight-week follow-up examinations. The proportion of perceived improvement of coldness was calculated and compared between the intervention and control groups by chi-square test. The proportion of perception of improved peripheral coldness in the intervention and control groups were calculated and compared by *t*-test.

At the baseline examination, two women in the intervention group had higher blood flow values at 10 minutes after the cold bathing test as compared to the value before the test (percentage recovery blood flow >300%). These two subjects were excluded for the sensitivity analysis. Also, since smoking is one of the strong factors for vasoconstriction, we conducted another sensitivity analysis by excluding two current smokers in the intervention group, and one smoker in the control group.

The 9.2 version of SAS was used for all statistical analyses. All statistical tests were two-tailed, and p < 0.05 was regarded as statistically significant.

## Results

Table 2 shows the mean values and proportions of the baseline characteristics of the participants. There were no differences between the intervention and control groups at the baseline in terms of age-adjusted mean values of body mass index, systolic and diastolic blood pressures, selected blood chemistry and hematology, peripheral blood flow before bathing, skin temperature, and age-adjusted proportions of peripheral body chilliness, peripheral coldness related symptoms, current and past smoking habits, exercise habits, perceived mental stress, use of antihypertensive medications and menopausal status.

**Table 2:** **Mean values ± standard deviations and proportions of baseline characteristics in the intervention and control groups at the baseline examination**

**Baseline variables**	**Intervention**	**Control**	**p value**
Number	28	30	
Age, year	49.4 ± 14.8	50.1 ± 13.2	0.86
Body mass index, kg/m^2^	20.6 ± 2.5	20.4 ± 3.0	0.72
Current smokers, %	7.1	3.3	0.52
Past smokers, %	14.3	16.7	0.81
Ethanol intake, g/day	2.7 ± 6.4	3.1 ± 6.5	0.80
Habitual exercise, %	67.9	56.7	0.39
Perceived mental stress, %	39.3	43.3	0.76
Systolic blood pressure, mm Hg	111.1 ± 17.4	106.9 ± 13.0	0.29
Diastolic blood pressure, mm Hg	66.3 ± 10.2	62.8 ± 6.4	0.12
Medication use for hypertension, %	10.7	3.3	0.28
Total cholesterol, mg/dl	209.3 ± 27.3	213.0 ± 42.3	0.69
LDL-cholesterol, mg/dl	117.9 ± 25.2	124.2 ± 37.8	0.46
HDL-cholesterol, mg/dl	72.7 ± 12.5	70.8 ± 14.2	0.60
Triglycerides, mg/dl	78.9 ± 42.3	71.2 ± 28.8	0.42
Blood glucose, mg/dl	92.3 ± 4.8	91.7 ± 7.4	0.76
hs C-reactive protein, mg/L	44.6 ± 47.7	56.2 ± 151.7	0.70
Red blood cell counts, ×10^4^/mm^3^	404.7 ± 24.7	409.5 ± 32.1	0.53
Hemoglobin, g/dl	12.8 ± 1.2	12.8 ± 0.8	0.99
White blood cell counts ×10^2^/mm^3^	49.0 ± 13.5	43.7 ± 10.8	0.11
Menopause, %	42.9	46.7	0.78
Fatigue of the eye, %	82.1	66.7	0.18
Dizziness, %	50.0	36.7	0.31
Ear ringing, %	25.0	16.7	0.65
Headache, %	39.3	33.3	0.64
Shoulder stiffness, %	78.6	83.3	0.65
Hot flash, %	28.6	36.7	0.52
Cough, %	32.1	26.7	0.65
Diarrhea, %	25.0	13.3	0.26
Constipation, %	57.1	33.3	0.07
Pain in knee or lower back, %	64.3	73.3	0.46
Palpitation, %	35.7	20.0	0.18
Short of breath, %	35.7	33.3	0.85
Swelling of lower extremities and/or eyelids, %	64.3	43.3	0.11
Finger chilliness, %	64.3	53.3	0.41
Foot chilliness, %	67.9	70.0	0.86
Back chilliness, %	46.4	36.7	0.46
Whole body chilliness, %	10.7	10.0	0.93
Peripheral blood flow, PU^a)^	81.9 ± 44.5	78.8 ± 52.8	0.82
Skin temperature, °C	29.9 ± 1.24	29.6 ± 1.43	0.61

a) PU: perfusion units = (number of moving blood cells in the measured contents) × (average velocity of blood cells).

Table 3 shows the peripheral blood flow and Table 4 shows the skin temperature of the participants at the baseline examination in January, and at the eight-week follow-up examination in March 2010 and 2011, for all participants (n = 58) and for participants without an abnormally high percentage recovery of blood flow (n = 56) at the baseline. Mean values of the percentage recovery of peripheral blood flow for all the participants in the intervention and control groups at the baseline examination were 13.3% and −27.8%, respectively (p for difference = 0.07), and those of the skin temperature were −9.6% and −13.5% (p = 0.04), respectively. Mean value of the percentage recovery of peripheral blood flow after the TJ-38 treatment in the intervention group was 17.2%, whereas it remained at −28.2% in the control group (p for difference = 0.007). However, mean value of percentage recovery of skin temperature after the treatment did not differ between the intervention and control groups. When the two women with percentage recovery of peripheral blood flow >300% at the baseline examination were excluded, the results did not change materially. Mean values of percentage recovery of peripheral blood flow were −13.1% in the intervention group and −27.8% in the control group (p for difference =0.29) at the baseline examination and 17.7% and −28.2%, respectively (p for difference = 0.008) at the follow-up examination.

**Table 3:** **Mean values ± standard deviations of blood flow in the intervention and control groups at the baseline and at the eight-week follow-up examinations**

	**Baseline**			**Follow-up**		
	**Intervention**	**Control**	**p value**	**Intervention**	**Control**	**p value**
Total participants						
Number	28	30		27	30	
Blood flow before bathing, PU	81.9 ± 44.5	78.8 ± 52.8	0.82	102.1 ± 60.3	101.5 ± 64.9	0.97
Blood flow 1 min after bathing, PU	50.7 ± 23.0	47.6 ± 23.2	0.61	59.1 ± 27.2	61.1 ± 37.3	0.82
Blood flow 10 min after bathing, PU	91.8 ± 85.0	51.0 ± 46.3	0.03	107.8 ± 71.4	80.1 ± 75.2	0.16
Percentage recovery of blood flow after 10 min, %	13.3	−27.8	0.07	17.2	−28.2	0.007
Participants without percentage recovery of peripheral blood flow >300% at the baseline (sensitivity analysis)						
Number	26	30		25	30	
Blood flow before bathing, PU	84.0 ± 45.1	78.8 ± 52.8	0.70	103.9 ± 62.3	101.5 ± 64.9	0.89
Blood flow 1 min after bathing, PU	51.0 ± 23.1	47.6 ± 23.2	0.59	59.6 ± 27.9	61.1 ± 37.3	0.87
Blood flow 10 min after bathing, PU	80.7 ± 75.1	51.0 ± 46.3	0.08	109.2 ± 73.6	80.1 ± 75.2	0.16
Percentage recovery of blood flow after 10 min, %	−13.1	−27.8	0.29	17.7	−28.2	0.008

**Table 4:** **Mean values ± standard deviations of skin temperature in the intervention and control groups at the baseline and at the eight-week follow-up examinations**

	**Baseline**			**Follow-up**		
	**Intervention**	**Control**	**p value**	**Intervention**	**Control**	**p value**
Total participants						
Number	28	30		27	30	
Skin temperature before bathing, °C	29.9 ± 1.24	29.6 ± 1.43	0.43	30.1 ± 1.35	29.9 ± 1.71	0.56
Skin temperature 1 min after bathing, °C	24.0 ± 0.83	23.7 ± 0.80	0.32	24.0 ± 0.99	23.9 ± 1.09	0.89
Skin temperature 10 min after bathing, °C	27.0 ± 2.79	25.6 ± 2.14	0.03	27.6 ± 2.72	26.5 ± 3.01	0.17
Percentage recovery of skin temperature after 10 min, %	−9.6	−13.5	0.04	−8.5	−11.4	0.14
Participants without percentage recovery of peripheral blood flow >300% at the baseline (sensitivity analysis)						
Number	26	30		25	30	
Skin temperature before bathing, °C	29.9 ± 1.26	29.6 ± 1.43	0.34	30.2 ± 1.40	29.9 ± 1.77	0.54
Skin temperature 1 min after bathing, °C	24.0 ± 0.77	23.7 ± 0.80	0.18	24.0 ± 1.03	23.9 ± 1.09	0.89
Skin temperature 10 min after bathing, °C	26.8 ± 2.74	25.6 ± 2.14	0.08	27.6 ± 2.83	26.5 ± 3.01	0.20
Percentage recovery of skin temperature after 10 min, %	−10.7	−13.5	0.11	−8.7	−11.3	0.19

When current smokers (2 in the intervention and 1 in the control groups) were excluded, there were no major difference in the results; mean values of percentage recovery of blood flow were 16.1% in the intervention group and −25.8% in the control group (p for difference = 0.08) at the baseline examination, and 23.2% and −26.7%, respectively (p for difference = 0.004) at the follow-up examination.

Table 5 shows the number and proportion of women in the intervention and control groups whose percentage recovery of blood flow after 10 min was >50% at the baseline and follow-up examinations. The proportion of women with >50% increase in the percentage recovery of peripheral blood flow increased in the intervention group (from 21% to 32%), whereas it decreased in the control group (10% to 0%) (p = 0.0007 at the follow-up examination). The sensitivity analysis excluded the two women with percentage recovery of peripheral blood flow >300% at the baseline examination showed similar results: 15% to 35% in the intervention group, and 10% to 0% in the control group (p for difference =0.0004 at the follow-up examination). When current smokers were excluded, there were no major difference in the results; the proportion of women with >50% increase in the percentage recovery of peripheral blood flow was 23% in the intervention group and 10% in the control group (p for difference = 0.21) at the baseline examination, and 35% and 0%, respectively (p for difference = 0.0005) at the follow-up examination.

**Table 5:** **Number and proportion of women whose percentage recovery of blood flow after 10 min was >50%, for the intervention and control groups at the baseline and at the eight-week follow-up examinations**

	**Baseline**			**Follow-up**		
	**Intervention**	**Control**	**p value**	**Intervention**	**Control**	**p value**
Total participants						
Number	28	30		27	30	
Percentage recovery of blood flow after 10 min >50%	6 (21%)	3 (10%)	0.23	9 (32%)	0 (0%)	0.0007
Participants without percentage recovery of peripheral blood flow >300% at baseline (sensitivity analysis)						
Number	26	30		25	30	
Percentage recovery of blood flow after 10 min >50%	4 (15%)	3 (10%)	0.54	9 (35%)	0 (0%)	0.0004

Proportions of the percentage recovery of blood flow after 10 min that were >50% are shown in parentheses.

The perception of improved peripheral coldness was reported by 81% of the women in the intervention group and 13% of the women in the control group (p < 0.001). Women who felt an improvement in peripheral coldness tended to have the greater mean value of the percentage recovery of blood flow than participants who felt otherwise: 9% in the intervention group and −20% in the control group (p for difference = 0.08 at the follow-up examination). The results did not change materially when we excluded the two women with percentage recovery of peripheral blood flow >300% at the baseline examination: 9% in the intervention group and −20% in the control group (p for difference = 0.10). When current smokers were excluded, there were no major difference in the results; 15% in the intervention group and −18% in the control group (p for difference = 0.06).

## Discussion

In the present parallel-group randomized controlled trial, women in the intervention group showed significant recovery of peripheral blood flow in the finger after the cold bathing test, as well as subjective improvement of peripheral coldness, whereas women in the control group experienced reduced recovery of peripheral blood flow and less subjective improvement. The exclusion of the two women (non-smokers) with an abnormally high percentage recovery of peripheral blood flow in the intervention group at the baseline examination did not alter the result, and supported the evidence of improvements in the recovery of peripheral blood flow and in peripheral coldness. The percentage recoveries of peripheral blood flow at the baseline examination for these two women were 307% and 406%, and at follow-up examination 29% and −7.6%, respectively. Both women reported subjective improvement in peripheral coldness. No other participants from either the intervention or control group showed a percentage recovery of peripheral blood flow >300% (at most 227%) at the follow-up examination. Therefore, the very high percentage recoveries of peripheral blood flow at the baseline examination were likely to be outliers, and were probably due to measurement error.

A randomized controlled trial of 72 children (39 boys and 33 girls aged 4 to 18 years) experiencing peripheral coldness showed that subjective improvement of symptoms was observed in 94% of the TJ-38 treatment group, whereas it was 54% for the vitamin E treatment group and 20% for the control group [18]. Another non-randomized trial involving 70 male patients who were chainsaw users with peripheral blood flow disturbance showed that the percentage recovery of skin temperature after the cold bathing test was larger in the group with TJ-38 treatment and physiotherapy, compared to that of the group treated with physiotherapy alone [19].

Our study is the first to analyze the effects of TJ-38 on peripheral coldness using an objective index (peripheral blood flow) and a subjective criterion (improvement of peripheral coldness) in a randomized controlled trial. Therefore, our study provides robust evidence of the effectiveness of TJ-38 in improving peripheral coldness.

The mechanism of TJ-38 on peripheral coldness is not well established. One of the pharmacological effects of TJ-38 may be the activation of autonomic nerve function. A study of eight healthy volunteers showed that plasma noradrenalin concentrations increased, and that the Low Frequency/High Frequency ratio in heart rate variability, an index of parasympathetic nerve activity, decreased two hours after the administration of TJ-38 [20]. Another study reported that evodiamine (in *Goshuyu*, an ingredient of TJ-38), a chemical extracted from *Evodia fruit* in the Tetradium family of plants, is an agonist for the transient receptor potential cation channel, subfamily V, member 1 (TRPV1), which promotes nitric oxide production in endothelial cells, and thereby relaxes the smooth muscles of vessels [13]. An extract from Paeoniae Radix (*Syakuyaku*, another ingredient of TJ-38) has vasodilatory and anticoagulant effects [14]. Adenosine, ligustilide, and butylidenephthalide from Angelica acutiloba, another ingredient of TJ-38, were reported to have an inhibitory activity against platelet aggregation [21,22]. These vasodilator and anticoagulation effects may contribute to the improvement in peripheral blood flow and peripheral coldness.

The limitations of this study are as follows: Firstly, subjects in the control group did not take placebo in this trial because it is very difficult and in many cases impossible to produce placebo for its unique smell and taste. However, measurement of percent recovery of peripheral blood flow was an objective measure, was blind for intervention or control. No placebo control unlikely lead to the biased results. Secondly, the measurement errors for the peripheral blood flow and skin temperature were inevitable. In a previous study [23], the coefficient of variation of blood flow (Periflux Systems) of five subjects on five different days within 2-3 weeks was 20-21%. In the present trial, however, two women in the intervention group showed an abnormally high percentage recovery of peripheral blood flow at the baseline examination. That potential measurement error might reduce the comparability of the mean percentage recovery of peripheral blood flow at the baseline examination between the two groups, although the difference did not reach statistical significance. Thirdly, we did not evaluate the vasodilatory and anticoagulation effects of TJ-38 directly because of the technical difficulty. That evaluation, however, would have made the mechanism clearer.

## Conclusions

We demonstrated that eight-week administration of TJ-38, a traditional herbal medicine, improved peripheral blood flow and perception of peripheral coldness in women experiencing peripheral coldness in a randomized controlled trial.

## References

[1] Takeuchi T, Nakao M, Kohno M, Hatano M, Niimi M, Yano E (2008). Development of a questionnaire to assess ‘Hie’ symptoms using an evidence-based analysis. Environ Health Prev Med.

[2] Kondo M, Okamura Y (1987). Cold constitution: analysis of the questionnaire (in Japanese with English abstract). Nihon Sanka Fujinka Gakkai Zasshi.

[3] Miyamoto N, Aoki T, Muto N, Inaba R, Iwata H (1995). Relationship between chilliness of the limbs and daily-life conditions in young females (in Japanse with English abstract). Nihon Eiseigaku Zasshi.

[4] Miyazaki J, Taniguchi T, Kuge H, Takeda T, Fujikawa A, Morisawa T (2009). Sex specific relationship between body mass index and health related QOL among persons with feeling of chillness –comparison with and without feeling of chillness- (in Japanese). QOL J.

[5] Takatori A, Okuda H, Sekiba K, Tanizaki K (1990). Thermological study on the coldness in women (in Japanese). Papers Institute Environ Med Okayama Univ Med School.

[6] Ushiroyama T, Sakuma K, Nosaka S (2006). Comparison of effects of vitamin E and wen-jing-tang (unkei-to), an herbal medicine, on peripheral blood flow in post-menopausal women with chilly sensation in the lower extremities: a randomized prospective study. Am J Chinese Med.

[7] Ushiroyama T, Kajimoto Y, Sakuma K, Ueki M (2005). Assessment of chilly sensation in Japanese women with laser Doppler fluxmetry and acceleration plethysmogram with respect to peripheral circulation. Bull Osaka Med Coll.

[8] Okada M, Uno M, Nagano E, Nomura Y, Ohira T, Sato S (2005). The relation of cold-water loading thermography with physical findings and lifestyle habit among women who have a cold constitution (in Japanese with English abstract). Biomed Thermol.

[9] Takimoto H, Yoshiike N, Kaneda F, Yoshita K (2004). Thinness among young Japanese women. Am J Public Health.

[10] Collins EG, Edwin Langbein W, Orebaugh C, Bammert C, Hanson K, Reda D (2003). PoleStriding exercise and vitamin E for management of peripheral vascular disease. Med Sci Sports Exerc.

[11] Jorneskog G, Brismar K, Fagrell B (1995). Skin capillary circulation severely impaired in toes of patients with IDDM, with and without late diabetic complications. Diabetologia.

[12] Nilsson L, Apelqvist J, Edvinsson L (1998). Effects of alpha-trinositol on peripheral circulation in diabetic patients with critical limb ischaemia. A pilot study using laser Doppler fluxmetry, transcutaneous oxygen tension measurements and dynamic capillaroscopy. Eur J Vasc Endovasc Surg.

[13] Shoji N, Umeyama A, Takemoto T, Kajiwara A, Ohizumi Y (1986). Isolation of evodiamine, a powerful cardiotonic principle, from Evodia rutaecarpa Bentham (Rutaceae) (in Japanese with English abstract). J Pharm Sci.

[14] Nakamura T, Okamoto S, Saito Y, Kuwaki T, Matsumoto H (1983). A mechanism to inhibit plasmin and thrombin in Paeoniae radix (in Japanese). Blood Vessel.

[15] Yakubo S, Ueda Y, Tanekura N, Shimabukuro H, Arashima Y, Namiki T (2013). The effectiveness of the kampo preparation Toki-shigyaku-ka-goshuyu-shokyo-to for treatment of sensation of cold due to spinal cord infarction. Int Med J.

[16] Kimura Y, Tanaka A, Sato H (2012). Efficacy of kampo formula tokishigyakukagoshuyushokyoto for cold syndrome evaluated with a novel clinical method using a patient-based questionnaire database. Kampo Med.

[17] Nilsson GE (1984). Signal processor for laser Doppler tissue flowmeters. Med Biol Eng Comput.

[18] Mori S (1984). A case-control study for frostbite with Tokishigyakukagoshuyushokyoto (in Japanese). Kampo Shinryo.

[19] Iwata H, Kasamatsu T, Miyashita K, Oshima R, Yamashita T, Ichimiya G (1983). An effect of Tokishigyakukagoshuyushokyoto on peripheral blood flow (in Japanese). Shinryo Shinyaku.

[20] Yakubo S, Yagi H, Kanmatsuse K, Hanakawa K, Nakai T, Takase H (1997). Effects of Tokishigyakukagoshuyushokyoto on improvement of “Hie-sho” (in Japanese with English abstract). Wakan Iyaku Gakkaishi.

[21] Kosuge T, Ishida H, Yamazaki H, Ishi M (1984). Studies on active substances of the herbs used for oketsu, blood coagulation, in Chinese medicine. I. On anticoagulant activities of the herbs used for oketsu (in Japanese). Yakugaku Zassi.

[22] Kawashiri N, Toriizuka K, Adachi I, Ueno M, Terasawa K, Horikoshi I (1986). Effects of traditional crude drugs on fibrinolysis by plasmin: antiplasmin principles in eupolyphaga. Chem Pharm Bull (Tokyo).

[23] Sundberg S (1984). Acute effects and long-term variations in skin blood flow measured with laser Doppler flowmetry. Scand J Clin Lab Invest.

